# C-kit receptor immunopositive interstitial cells (Cajal-type) in the porcine reproductive tract

**DOI:** 10.1186/s13028-017-0300-5

**Published:** 2017-05-19

**Authors:** Malgorzata Domino, Bartosz Pawlinski, Romuald Zabielski, Zdzislaw Gajewski

**Affiliations:** 1 Department of Large Animal Diseases with Clinic, Faculty of Veterinary Medicine, Warsaw University of Life Sciences (WULS-SGGW), Nowoursynowska 100, 02-797 Warsaw, Poland; 2 Veterinary Research Centre and Center for Biomedical Research, Warsaw University of Life Sciences (WULS-SGGW), Nowoursynowska 100, 02-797 Warsaw, Poland

**Keywords:** Myometrium, Oviduct, C-kit/CD 117, Interstitial Cajal-like cell, ICLC, Sow

## Abstract

**Background:**

Interstitial Cajal cells have been suspected as being the pacemaker cells of smooth muscle motor activity and discharging slow triggering waves in the gut as well as in other organs containing smooth muscles where they are known as interstitial Cajal-like cells (ICLC). The present study describes ICLC localization and density in the porcine oviduct and uterus. Differences in ICLC density were examined using histological, immunohistochemical and immunofluorescent methods and c-kit expression was determined.

**Results:**

interstitial Cajal-like cells with characteristic morphological and immunological phenotypes were found. Star-like or spindle-shaped cells with very long, moniliform processes were localized in the muscle layers of the oviduct and uterine walls at variable densities that decreased progressively from high in the oviduct to low in the uterus.

**Conclusions:**

The detailed description of ICLC in the porcine reproductive tract may lead to a better understanding of reproductive tract motility. Our approach is inexpensive and effective for ICLC evaluation and may in the future be applied to clinical diagnosis.

## Background

Interstitial Cajal cells (ICC) are the pacemaker cells of gut motor activity [1]. They have been found in the wall of the gastrointestinal tract of several species, including humans [2, 3]. The ICC may be essential for smooth muscle contractility in the gastrointestinal tract and probably also in the urinary and reproductive tracts by discharging slow triggering waves [4]. More recently, cells with morphology and antigenicity similar to ICC have been found outside the gastrointestinal tract, i.e., in the vas deferens [5], urethra [6], bladder [7], prostate gland [8], lymphatic vessels [9], uterus [10], blood vessels [11], fallopian tube [12, 13], mammary gland [14] and pancreas [15] of mice, rats and humans. These cells are most commonly known as interstitial Cajal-like cells (ICLC). The defining features of ICLC were indicated by examination of hematoxylin and eosin (HE) stained sections, characterized by immunohistochemistry and confirmed by transmission electron microscopy (TEM) [13]. Ultrastructural criteria were proposed by Huizinga et al. [16] for the enteric ICC and by Popescu et al. [13] for extra-intestinal ICLC. ICLC ultrastructure provides valuable morphological hints for ICLC identification. TEM identifies mainly qualitative characteristics so Popescu et al. [13] performed immunohistochemical studies to specifically identify ICLC. They observed cells with typical interstitial localization and phenotype more or less similar to those of ICC. The discovery of the c-kit receptor as a marker for ICLC allows easier recognition of these cells at the light microscopic level. The type III tyrosine kinase receptor (c-kit; Kit protein; CD117) present on the cell surface is one of the criteria for specific identification of ICLC [17]. Because c-kit expression can also be detected in mast cells [18], morphological evaluation based on live-tissue methylene blue staining or HE staining of fixed tissues and assessment of ultrastructure by TEM or scanning electron microscopy (SEM) is crucial for proper identification of ICLC. The ICLC morphology is characterized as a triangular, spindle-shaped or star-like body, a distinct nucleus, and two or more, very long, moniliform processes [13, 18]. The ICLC display on TEM images as a slender cell body with a thin rim of cytoplasm around the nucleus and long cytoplasmic processes that suddenly emerge from the cellular body. Organelles are distributed near the nuclear pole and in the dilated regions of cell prolongations [13, 16].

The morphological and functional roles of ICLC in the reproductive tract have also been the subject of interest recently and c-kit positive cells have been identified in the human uterus [10]. Subsequently, ICLC have been described in the human uterus and fallopian tube [13]. Finally, re-investigation of cells originally regarded as being ICLC in the male and female reproductive tracts of mice, rats, rabbits and humans has identified them as ‘myometrial Cajal-like interstitial cells’ (m-ICLC) to describe their ultrastructural similarities with ICC [19]. These m-ICLC again seemed to be located mainly in the boundaries of smooth muscle bundles throughout the myometrium, forming a network between smooth muscle cells, nerves and capillaries [18], and were present in myometrial biopsies taken throughout reproductive life [19, 20]. A further study supported the observation that m-ICLC are c-kit positive [20]. Popescu’s team tested many markers and concluded that the positive reaction of markers differs between organs and species [14]. On the other hand, there is a lack of morphological and physiological evidence of ICLC in livestock species, especial within the reproductive tract.

The aim of the present study was to localize and identify ICLC in the porcine reproductive tract and describe their morphology in order to help understanding of reproductive tract motility. This may have clinical implications by explaining infertility in sows without any proven uterine and fallopian tube abnormalities.

## Methods

### Myometrial tissue samples

Full-thickness tissue samples of porcine myometrium were obtained at a slaughterhouse from 12 mature sows aged 10–24 months, body weight 80–140 kg. The animals were clinically healthy and their reproductive tracts did not show any gross pathology. To avoid endometrial contamination, parts of the external muscular layer of the uterus (body, middle of the horn, horn tip) and oviducts (isthmus, infundibulum) were excised from areas free of macroscopically visible abnormalities. All tissue samples were obtained in accordance with a protocol approved by the local Ethics Committee (No 10/2011), in accordance to generally accepted international practice.

### Histology

Unless otherwise indicated, all reagents were purchased from Sigma-Aldrich (Sigma-Aldrich, Poland). Tissue samples were fixed in 4% neutral buffered paraformaldehyde, processed, embedded in paraffin and 5 µm sections were prepared using standard histology procedures. Sections were mounted on glass slides, de-paraffinized and rehydrated in a series of xylene and decreasing concentrations of ethanol. Slides were then stained with HE using a standard protocol and mounted under Canadian balsam resin for histological evaluation. Immunohistochemical and immunofluorescence labeling against CD117 (c-kit) was performed using a staining protocol modified from Ciontea et al. [18]. For the assessment of immunohistochemical and immunofluorescence reactions for CD117 specificity, porcine ileum paraffin blocks were used as a positive control (PC) and staining protocols with the primary antibody omitted were used as a negative control (NC).

### Electron microscopy

Tissue samples were also frozen in liquid nitrogen using standard procedures. Afterwards, samples were cut using a cryostat to 15 µm sections. Sections were mounted on aluminum pin stubs using double-sided carbon adhesives. Fresh sections were examined under a SEM Phenom XL (Phenom-World, Netherlands).

### Immunohistochemistry

Prior to immunolabeling, slides were boiled in citrate buffer for antigen-retrieval, then washed in phosphate-buffered saline (PBS) and permeabilized in PBS containing 1% bovine serum albumin (BSA) for 30 min. Primary antibodies (CD117/c-kit, Dako, USA) were applied in a 1:100 dilution for 1 h at 4 °C. A HRP-labelled polymer conjugated with secondary antibodies and 3,3′-diaminobenzidine (DAB)+ substrate-chromogen [EnVision+ System-HRP (DAB), Dako, USA] were used to detect the primary immune reaction. Following incubation steps, slides were rinsed three times in PBS. After labeling, the coverslips were mounted with Canadian balsam. The slides were examined under a light microscope BX-61 (Olympus, Poland) using MicroImage, v. 4.0 software (Olympus, Poland). Negative controls were prepared following the same protocol, but omitting the primary antibodies.

### Immunofluorescence

Polyclonal Alexa Fluor 488 (Molecular Probes, USA) labeled chicken anti-mouse antibodies (dilution 1:500 for 2 h at 4 °C) were used to detect the primary immune reaction (the first part of the protocol was identical to the IHC protocol). Finally, nuclei were counterstained with 1 mM 7-Aminoactinomycin D (7-AAD, 250 μl/ml, Sigma Aldrich, Poland). Following incubation steps, samples were rinsed three times in PBS. After labeling, the coverslips were mounted using mounting medium for fluorescence microscopy. Immunofluorescence labeled cells were examined under a confocal microscope (FV-500, Olympus, Poland). Quantitative evaluation was performed under a scanning cytometer (SCAN^R, Olympus, Poland).

### Statistical analyses

The immunohistochemical and immunofluorescence data were statistically analyzed by Graph-Pad InStat software (San Diego, USA). A one-way analysis of variance (ANOVA) was performed, followed by the Tukey–Kramer Multiple Comparisons Test, with P < 0.005 considered significant and P < 0.0001 as highly significant.

## Results

**Fig. 1:**
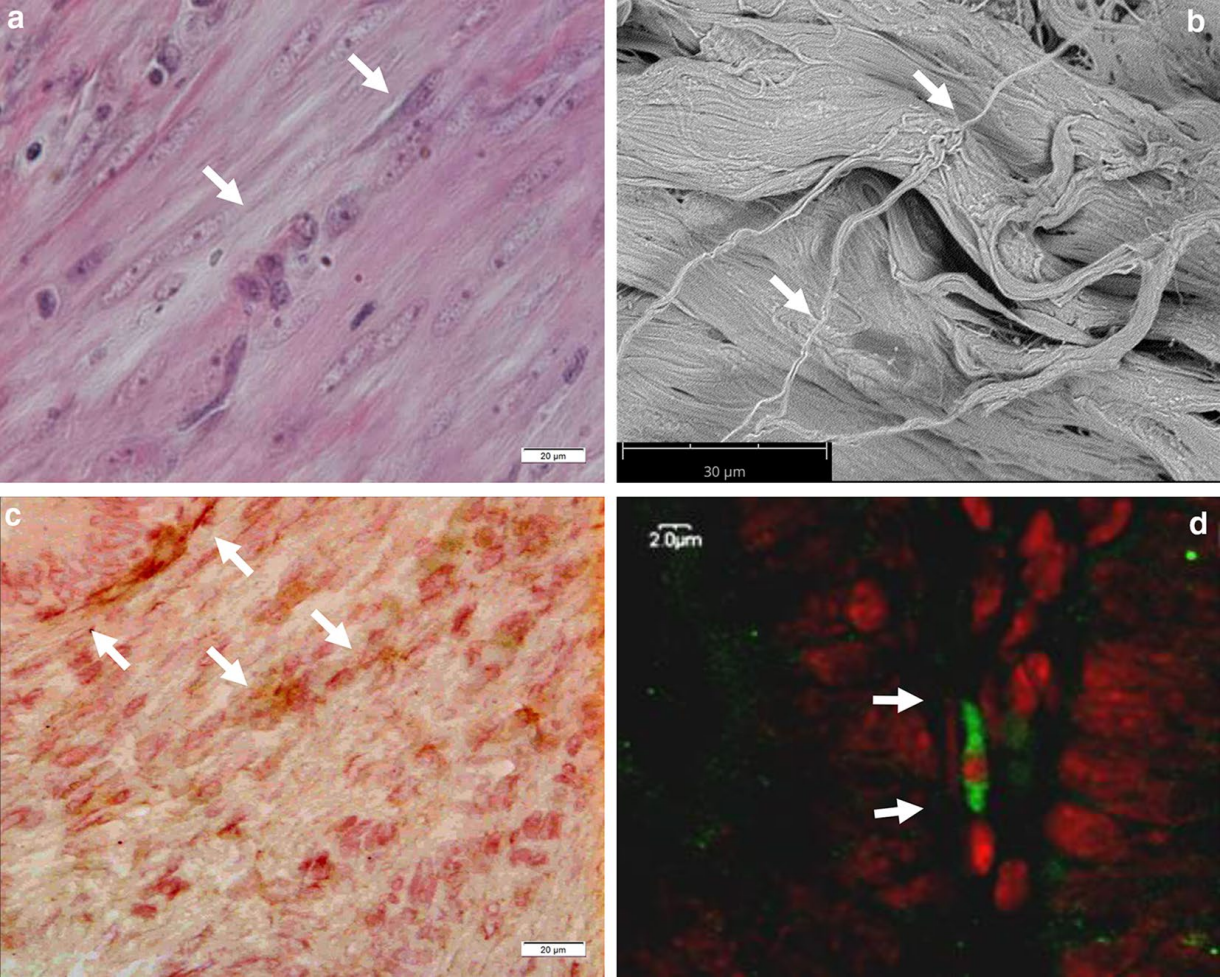
Muscular layer of the uterine body. Fusiform cells with long processes and oval nucleus show morphology typical for ICLC (*arrows*). **a** Wide-field microscopy of a HE stained tissue section, original magnification 400x. **b** Scanning electron microscopy, original magnification 1500x; **c** wide-field microscopy following IHC labeling, original magnification 400x; **d** confocal microscopy following IF labeling, original magnification 3000x

**Fig. 2:**
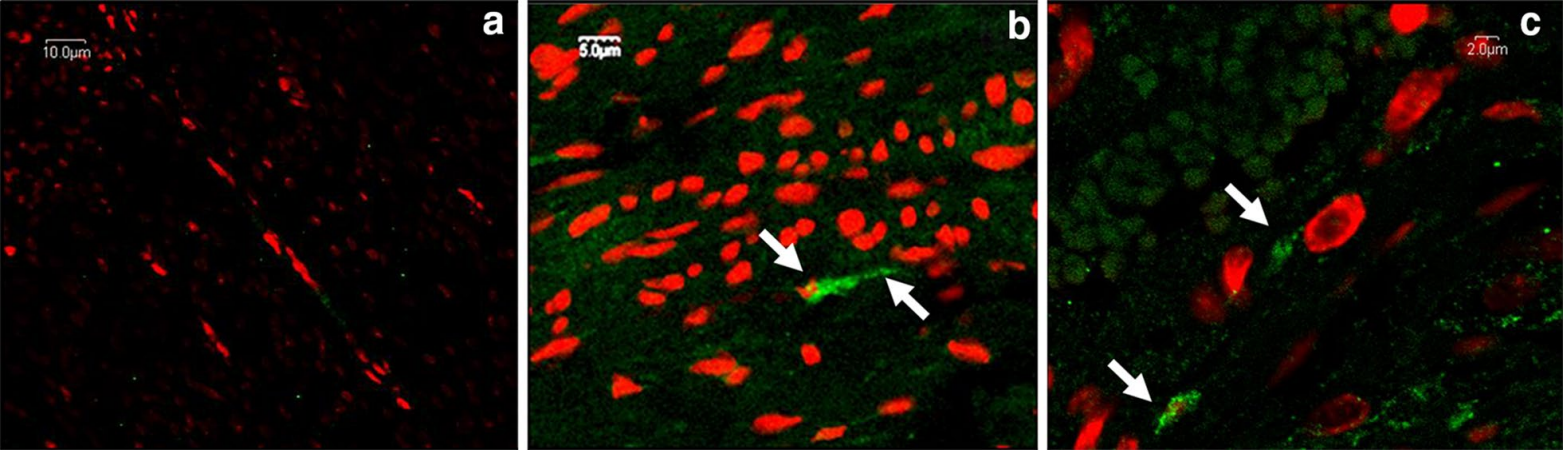
Confocal microscopy following IF labeling, emission spectra of AF 488 and 7AAD. **a** Muscular layer of corpus uteri—negative control (NC), original magnification 600x; **b** muscular layer of ileum—positive control (PC), original magnification 1500x; **c** muscular layer of corpus uteri—study group, original magnification 3000x

The characteristics of ICLC in the porcine reproductive tract were demonstrated by histology, immunohistochemistry, immunofluorescence and scanning electron microscopy (Fig. 1). ICLC were defined as c-kit-immunopositive nucleated cells with a triangular, spindle-shaped or star-like body and two or more, very long, moniliform processes. Such cells were also found in the positive control samples (porcine ileum) while they were absent in the negative controls (Fig. 2).

### ICLC evaluation

**Fig. 3:**
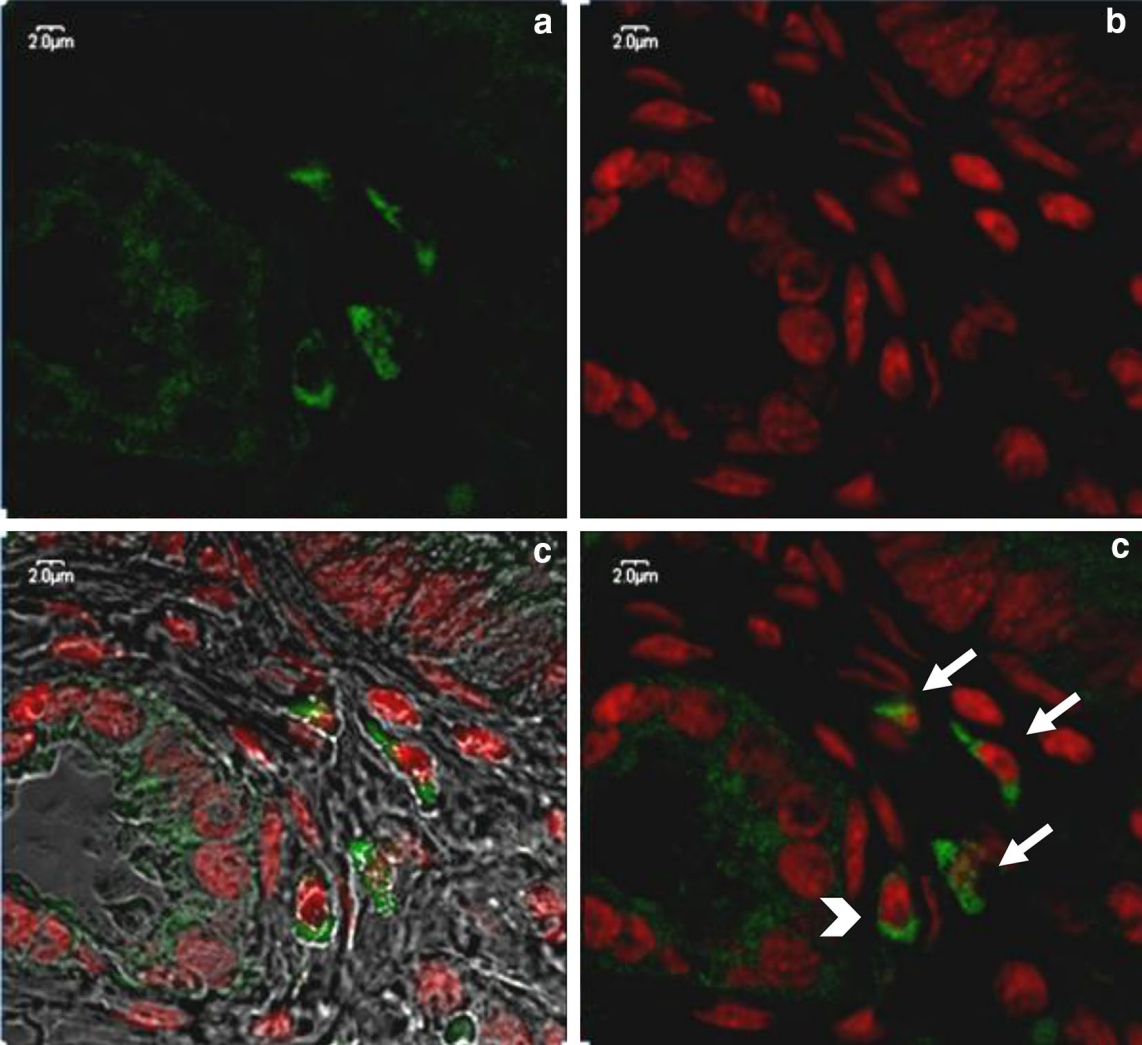
Muscular layer of oviduct infundibulum. A group of the fusiform and trigonal c-kit-positive cells (*arrows*) laid in close proximity, were distinguished from the c-kit-positive nonbranching mast cells (*arrowhead*). **a** Emission spectra of AF 488, **b** emission spectra of 7AAD, **c** emission spectra of AF 488 and 7AAD as well as tissue structure, **d** emission spectra of AF 488 and 7AAD. Confocal microscopy following IF labeling, original magnification 3000x

C-kit-positive ICC-like cells with dendritic processes and large and oval nuclei were identified in the uterine body, uterine horns (middle and tip) and oviducts (isthmus and infundibulum). They were distinguishable from the c-kit-negative and non-branching smooth muscle cells and from the c-kit-positive non-branching mast cells by confocal microscopy (Fig. 3). In the wide-field microscope, the basophil cytoplasm and large oval nucleus allowed ICLC to be distinguished from eosinophil myocytes and fibroblasts.

In both light and confocal microscopy, most ICLC displayed a characteristic morphology, i.e., a triangular, spindle-shaped or star-like body with two or more, very long, moniliform processes and a distinct nucleus. The typical ultrastructure of a triangular-shaped cell located between smooth muscle cells with electron dense cytoplasm, numerous mitochondria and tips of ICLC prolongations was demonstrated by SEM.

**Fig. 4:**
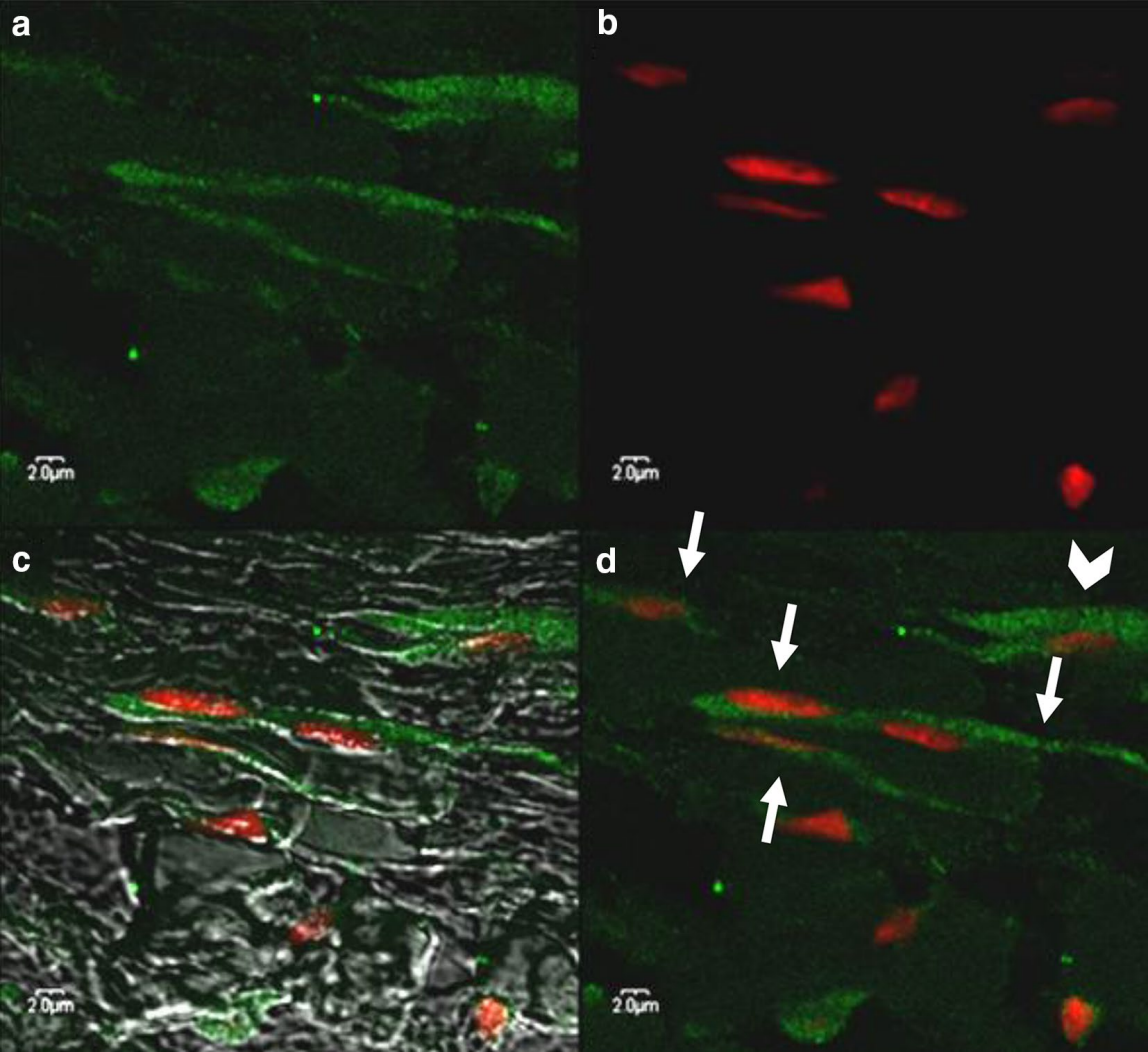
Muscular layer of tip of the uterine horn adjacent to the oviduct. Fusiform and trigonal c-kit-positive ICLC (*arrows*) as well as star-like shape ICLC (*arrowhead*) forming a network through the long cellular processes. **a** Emission spectra of AF 488, **b** emission spectra of 7AAD, **c** emission spectra of AF 488 and 7AAD as well as tissue structure, **d** emission spectra of AF 488 and 7AAD. Confocal microscopy following IF labeling, original magnification 3000x

The morphology of ICLC varied between different parts of the porcine reproductive tract. In the infundibulum and isthmus of the oviduct, ICLC consisted of fusiform cells with an oval nucleus and two cytoplasmic processes. In the uterine wall, however, the predominant type of ICLC were spindle-shape cells and triangular cells with three cytoplasmic processes. Star-like or pentagonal shaped cells were observed sporadically dispersed in the connective tissue layer, especially in the tip of the uterine horn adjacent to the oviduct (Fig. 4).

**Fig. 5:**
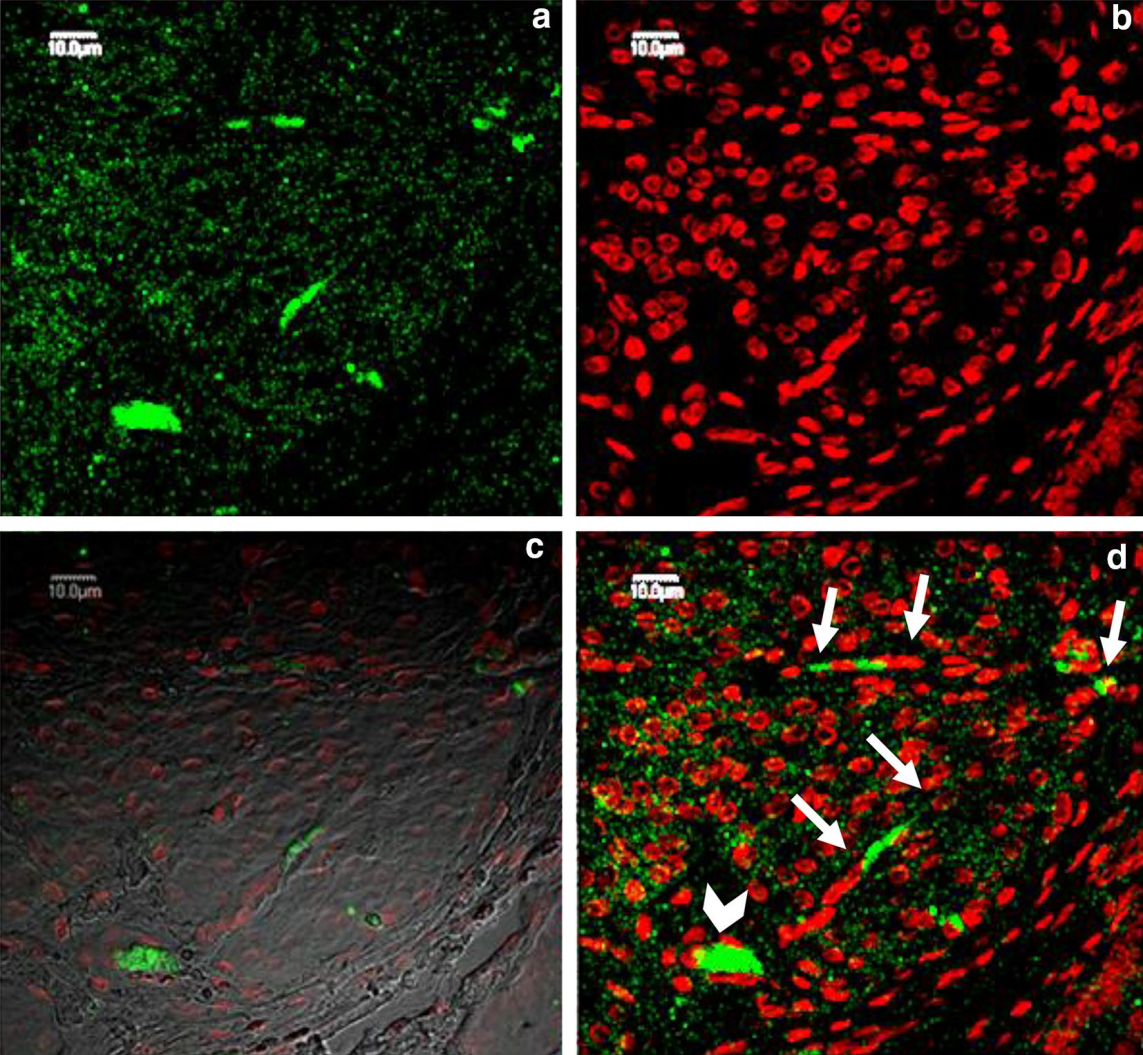
Muscular layer of tip of the uterine horn adjacent to the oviduct. Fusiform and trigonal c-kit-positive ICLC (*arrows*). The long, moniliform cellular processes formed a network contacting smooth muscle cells, nerve cells and capillaries (*arrowhead*). **a** Emission spectra of AF 488, **b** emission spectra of 7AAD, **c** emission spectra of AF 488 and 7AAD as well as tissue structure, **d** emission spectra of AF 488 and 7AAD. Confocal microscopy following IF labeling, original magnification 600x

In the uterus and oviducts, ICLC were predominantly located between smooth muscle cells of both longitudinal and circular muscle layers. Their processes formed a network connecting smooth muscle cells, nerve cells and capillaries (Fig. 5).

### ICLC density

The ICLC density differed among different parts of the reproductive tract (Tables 1 and 2).

**Table 1:** Interstitial Cajal-like cell density (mean ± SD) in segments of the porcine reproductive tract

Staining method	Oviduct (infundibulum)	Oviduct (isthmus)	Uterine horn (tip)	Uterine horn (middle)	Corpus uteri
HE	2.22 ± 0.09^a^	5.35 ± 0.80^b^	6.78 ± 0.76^c^	3.89 ± 0.44^d^	4.43 ± 0.79^d^
IHC	4.13 ± 0.35^A^	5.21 ± 0.31^B^	5.25 ± 0.24^B^	3.92 ± 0.25^A^	4.20 ± 0.45^A^
IF	2.16 ± 0.82^x^	4.12 ± 1.01^y^	3.10 ± 0.92^z^	3.17 ± 1.04^z^	1.22 ± 0.38^v^

*HE* hematoxylin-eosin staining, *IHC* immunohistochemical labeling, *IF* Immunofluorescent labeling

Kolmogorow-Smirnow test (α = 0.05). Density defined as cell numbers in 20 fields of view

Different letters indicate statistically significant differences independently in row HE, ICH, IF (P < 0.0001)

**Table 2:** Interstitial Cajal-like cells percentage (mean) in segments of the porcine reproductive tract

Staining method	Oviduct (infundibulum) (%)	Oviduct (isthmus) (%)	Uterine horn (tip) (%)	Uterine horn (middle) (%)	Corpus uteri (%)
HE	32.7^a^	79.0^b^	100.0^c^	57.4^d^	65.4^d^
IHC	78.6^A^	99.3^B^	100.0^B^	74.7^A^	79.9^A^
IF	52.4^x^	100.0^y^	75.3^z^	77.1^z^	29.6^v^

*HE* hematoxylin-eosin staining, *IHC* immunohistochemical labeling, *IF* immunofluorescence labeling

One-way ANOVA test with multiple comparisons Tukey test (P < 0.0001). Different letters indicate statistically significant differences

The highest density of ICLC was found in the isthmus of the oviduct and the uterine horn tip, while a relatively low density of ICLC was found in the uterus (horns and body). The ICLC density was on average 22–34% higher in the isthmus and the tip of the uterine horn adjacent to the oviduct than in the uterine horns and corpus, and the density decreased with increasing distance from the oviduct. However, in the oviduct itself, the ICLC density in the infundibulum was about 38% less than in the isthmus. A significantly higher (P < 0.0001) density of ICLC was found in the isthmus of the oviduct (5.21 ± 0.3; 99.3%) and in the uterine horn tip (5.25 ± 0.24; 100.0%) by immunofluorescence microscopy, while a lower density of ICLC was observed in the uterine horn (3.92 ± 0.25; 74.7%) and body (4.20 ± 0.45; 79.9%).

## Discussion

This study describes for the first time the occurrence and density of c-kit-positive ICLC in different parts of the porcine reproductive tract. Changes in ICLC density were estimated by histological, immunohistochemical and immunofluorescence methods and confirmed especially by scanning electron microscopy. The c-kit positive fusiform or spindle-shaped cells with multiple, long, thin, moniliform processes in a characteristic location (outside the epithelium) are in agreement with previous results [18, 19]. However, we did not find that routine HE staining was sufficient for identification of ICLC, as found also by Popescu et al. [13]. In our experience, the main problem remains the difficulty of differentiating between the cell processes and collagen fibers [12]. Hutchings et al. [19] assembled criteria that allowed the differentiation of m-ICLC from fibroblasts based on close contact with target nerve bundles and smooth muscle cells, characteristic cytoplasmic processes, and gap junctions with smooth muscle cells or with each other. Evaluation of these criteria in porcine tissues was possible when they were examined by immunofluorescence. Therefore, the HE approach was used only for initial screening and to examine tissue architecture.

The specificity of immunoreactions was confirmed in the positive control—porcine ileum used as reference material and the negative control—lack of signal. We confirmed similar immunophenotypes of ICC and ICLC in porcine specimens and ruled out detected signals as being from specific secondary antibody binding. Furthermore, the porcine ICLC immunophenotype was obtained by correlating morphology with immunohistochemistry and immunofluorescence. The specificity of the primary antibody (CD117/c-kit) was confirmed in porcine specimens using commercial antibody (Dako, USA). Popescu et al. [13] referred this method as the most effective. Those authors assessed the intensity of ICLC reactivity in tissues from the human uterus and fallopian tube and stated that the anti CD117/c-kit polyclonal antibody (Dako, USA) in 1:100 dilution demonstrated a strong reaction (i.e., strikingly positive even at low magnification). We adopted the published staining protocol for porcine tissues using both immunohistochemical and immunofluorescence labeling.

Interstitial Cajal-like cells have specific ultrastructural morphological features evident in transmission electron microscopy (TEM) that together with morphology and c-kit expression are considered the ‘gold’ [16] and ‘platinum standard’ [13] for identification of these cells. TEM of ultra-thin myometrium sections is not effective and is useless for routine clinical diagnosis. TEM requires extensive sample preparation in which the thickness of the specimens should be less than 100 nm. In comparison, sample preparation for SEM is not that difficult. SEM focuses on the sample’s surface and its composition whereas TEM provides details about internal composition. Therefore, TEM can show many more characteristics of the sample, such as morphology, crystallization, stress or even magnetic domains. On the other hand, SEM shows only the morphology of samples, which is enough for ICLC confirmation in screening sections but insufficient for detailed cell research (e.g., verifying multicontact synapses, distances between membranes, conformation of the endoplasmic reticulum). The SEM is a better tool for surface characterization compared to TEM which is better for internal structure analysis but it is still hardly effective for routine clinical use. Thus, we were looking for a simple, quick, relatively inexpensive and effective ICLC evaluation protocol that would be useful in clinical diagnosis of reproductive disorders in livestock animals. The protocols described in this study are easy to perform and permits general characterization of myometrial ICLC.

In agreement with other reports [20–23], the m-ICLC observed in porcine reproductive tract were c-kit positive. In all porcine oviductal and uterine slides the ICLC were located mainly on the boundaries of smooth muscle bundles throughout the myometrium, similar to human tissues [19], but the morphology and density were slight different, depending on cell localization. Similar to the human fallopian tube [12, 19] and myometrium [18, 19], cells with very long, moniliform cytoplasmic processes predominantly exhibiting fusiform and spindle-shaped or triangular cell bodies, respectively.

At the same time, a large number of ICLC was localized near small blood vessels which corroborates earlier findings [10, 12, 13, 23]. Shafic et al. [10] suggested that the ICC themselves are under the control of excitatory or inhibitory transmitters. Their close contact with blood vessels suggests that ICLC may be particularly responsive to humoral stimuli, which is supported by the presence of ICLC ovarian steroid hormone receptors. It was reported that human myometrial ICLC express estrogen and progesterone receptors [23, 24]. However, more studies are needed to fully elucidate the function of ICLC in the porcine reproductive tract.

Furthermore, in the present study, the highest density of ICLC was found in the isthmus of the oviduct and the tip of the uterine horn, while a lower density of ICLC was found in the uterus proper (horns and corpus). Therefore, based on quantitative analysis, we suggest the uterine horn tip as the place with the highest ICLC density. Shafic et al. [10] reported that uterine electric activity appears to be controlled through ICC, whose continuous pacemaker activity is conducted to the smooth muscle cells of the uterus. We speculate that ICLC accumulation in the isthmus of the oviduct and the tip of the uterine horn may be connected with ICLC functional activity. However, the role of ICLC as the “primary” pacemakers, which initiate slow waves in the myometrium and oviduct muscle layers, needs to be determined in future investigations.

## Conclusions

The results confirm the presence of ICLC in the porcine reproductive tract and indicate that a combination of light and confocal microscopy techniques is a viable approach to identify ICLC in clinical diagnostics. These methods are inexpensive and effective in ICLC evaluation. We believe that in the future our approach can be applied to clinical diagnosis. The detailed description of ICLC in the porcine reproductive tract may lead to a better understanding of reproductive tract motility and have clinical implications by explaining infertility in sows without any proven uterine and fallopian tube abnormalities.
